# CREATIVE STORIES FROM A MEMORY CARE COMMUNITY: VALUES, NORMS, IDENTITIES, AND EXPERIENCES

**DOI:** 10.1093/geroni/igz038.3202

**Published:** 2019-11-08

**Authors:** Kyong Hee Chee, Olga Gerhart, Seoyoun Kim, Sara Caldwell

**Affiliations:** 1 Texas State University, San Marcos, Texas, United States; 2 Texas State University, San Marcos, United States

## Abstract

As an arts-based, creative storytelling program for persons living with Alzheimer’s Disease and related dementias (ADRD), TimeSlips involves a facilitator showing a picture to participants, who then exercise their imagination to create a story. The program has shown to benefit participants’ well-being, possibly because of the opportunity to express themselves. Although they may reflect participants’ values and identities, the content of such stories had not been the focus of investigation. The aim of this study is, therefore, to identify major themes of such stories through a qualitative content analysis. We implemented a creative storytelling program at Silverado Onion Creek Memory Care Community (currently, The Auberge) in Austin, and offered 6 weekly sessions with 4 small groups of residents. A total of 26 residents participated in the study, creating 24 collective stories in total. Three researchers first open-coded these stories and then met to reach consensus concerning the themes that emerged. Ten themes were identified: family values, generativity, religious reference, reference to love, reference to home, cultural norms, uncertainty and worries, positivity, negativity, and dissonance and disagreements. The first 6 themes represent the values, beliefs, and norms of the participants, with the remaining 4 reflecting their personal identities, personalities, and experiences. The findings suggest that they continue to value families and religion, care about others, and make judgements about people, things, and circumstances that they face. Researchers, practitioners, and care partners can benefit from “listening to” creative storytellers more closely to learn about their opinions, expectations, and preferences.

